# *In vitro* reconstitution of kallikrein-kinin system and progress curve analysis

**DOI:** 10.1042/BSR20221081

**Published:** 2022-10-18

**Authors:** Bertrand Favier, Dominique J. Bicout, Rémi Baroso, Marie-Hélène Paclet, Christian Drouet

**Affiliations:** 1 Univ. Grenoble Alpes, CNRS, UMR 5525, VetAgro Sup, Grenoble INP, TIMC, 38000 Grenoble, France; 2 Univ. Grenoble Alpes, CNRS, UMR 5525, VetAgro Sup, Grenoble INP, CHU Grenoble Alpes, TIMC, 38000 Grenoble, France; 3 Institut Cochin, Equipe Batteux-Allanore, Université Paris Cité, INSERM UMR1016, Paris, France

**Keywords:** bradykinin, enzyme kinetics, kallikreins, serine proteases

## Abstract

Human kallikrein-kinin system (KKS) is a proteolytic cascade with two serine-protease zymogen couples (Factor XII and prekallikrein (PK) and their activated forms, FXIIa, PKa, respectively), releasing bradykinin by cleavage of native high-molecular-weight kininogen (nHK) into cleaved HK. For KKS investigation in human plasma, this cascade is usually triggered on ice eventually by mixing with purified proteins. It has been established that purified FXIIa, PK, and nHK required a fixed order and timing for mixing protein on ice to ensure reproducibility of testing, we investigated the activation kinetics of both enzymes. The activation process of this *in vitro* minimal reconstitution of KKS was studied by progress curve analysis, in condition of high enzyme/substrate ratio and by using on natural rather than peptide substrates.

FXIIa and PKa were found five-times less active on ice than at 37°C: *k*_cat_ = 0.133 ± 0.034 and 0.0119 ± 0.0027 s^−1^, *K*_M_ = 672 ± 150 and 115 ± 24 nM, respectively. The progress curve analysis of our *in vitro* KKS reconstitutions differed from a Michaelis–Menten mathematical simulation by a faster initial rate and a slower late rate. These two features were also observed *ex vivo* by using dextran sulfate-activated plasma and could reinforce the hypothesis of a maximal local effect (bradykinin release) and a minimal systemic consequence (PK preservation) in KKS activation process.

Analyzing the complete curve of cold KKS activation would provide valuable information for *ex vivo* investigation of KKS in samples from patients presenting with hereditary angioedema and other inflammatory conditions.

## Introduction

Many plasma serine proteases are produced from a zymogen, as inactive precursor, which can be activated by proteolytic cleavage [2]. Such proteolytic cascades are found in numerous biological processes, from embryonic development, arthropod innate immunity, to coagulation, complement, fibrinolysis in mammals [3,4]. Serine protease catalytic reaction, among the best documented enzymatic reactions [3,5], involves a catalytic triad and is described by three steps with (i) enzyme substrate equilibrium, (ii) acylation of the serine active site, and (iii) deacylation of the acyl enzyme [5]. The relation between the involved rate constants and the Michaelis–Menten (MM) constant (*K*_M_) and the turnover number (*k*_cat_) remains partly established [5]: in particular ‘acylation is rate determining for amide peptide substrates and deacylation for ester substrates,’ but the reverse is also supported by presteady-state burst observations from assays using amide peptides [5]. Protein substrates have scarcely been investigated.

Human kallikrein-kinin system (KKS) is a proteolytic cascades including two serine protease zymogens plus one protein substrate [4]. The first component, the Factor XII zymogen, is activated into the FXIIa serine protease upon a triggering event, for instance, after contact with polyanionic surface, and also participates in the extrinsic pathway of the coagulation cascade [6]. The second component is the prekallikrein (PK) zymogen, whose enzymatic cleavage by FXIIa generates plasma kallikrein (PKa), as second serine protease [4]. PKa has several substrates, including FXII, which is thus activated in an amplification feedback loop, and the native high-molecular-weight kininogen (nHK) [6]. Cleavage of nHK produces a cleaved species (cHK) and bradykinin (BK), a short-lived nonapeptide representing a potent vasodilator and inflammatory mediator [4]. KKS is under the control of several serpins (serine protease inhibitors), the main being C1 Inhibitor (C1-INH) [4]. Its encoding gene, *SERPING1*, can be affected by more than 800 variants leading to uncontrolled pathological BK release responsible of subsequent increased endothelial permeability and a clinical phenotype of hereditary angioedema (HAE) [7,8]. One of the key questions in HAE is the relationship between the hour-long angioedema effect of the short-lived BK production and the systemic plasmatic changes in the KKS activation process: the explanation might come from the cellular distribution of BK/*des*Arg^9^-BK receptors and of membrane peptidases, and also from the persistence of circulating activated proteases [7].

KKS is also at the cross-roads of many physiological pathways, including complement, coagulation, fibrinolysis, and renin-angiotensin system, extending KKS interest far beyond HAE field [4,9]. Major interests currently focus on a control/inhibition of KKS by drugs [10]. This highlights the need of an enzymatic model of KKS cascade activation.

While devising several tests to explore KKS components in plasma samples [11], we observed that purified FXIIa, PK, and HK required a fixed order and timing for mixing protein on ice to ensure reproducible data. The kinetics of substrate cleavage by PKa at 25°C [12] and by FXIIa at 37°C [13] have been established, but not those at 0°C, although KKS activity has already been measured at this temperature [14]. While a mathematical model of the positive feedback loop has been developed [6], the transformation of a two-step proteolytic cascade into a mathematical model remains elusive.

The present study aimed to investigate the kinetics of FXIIa and PKa at 0°C and at 37°C, and the KKS activation *in vitro*, using high enzyme/substrate ratio and focusing on natural substrate rather than peptide substrates. We used progress curve analysis instead of initial steady-state velocity [15]. A simulation of the progression using two MM-formatted differential equations indicated that KKS enzymatic parameters recorded at 0°C *in vitro* deviated from a MM mechanism by a faster initial rate and a slower late rate, features that have also been apparent in *ex vivo* investigations.

## Materials and methods

### Reagents

Purified nHK, PK, and FXIIa were purchased from Enzyme Research (Swansea, U.K.) or Calbiochem (Darmstadt, Germany), PKa from Calbiochem, Corn Trypsin Inhibitor (CTI) from ChemCruz (California, U.S.A.). After reconstitution according to the manufacturer, proteins were aliquoted in low binding polypropylene tubes and kept at –80°C. Protein concentrations were measured by bicinchoninic assay (microBCA; Interchim, Montluçon, France) by performing three independent 1/100 dilutions directly into the working 96-well plates. FXIIa, PK, and HK integrity was checked by western blot with the corresponding antibody.

Peroxidase-conjugated anti-C1-INH polyclonal antibody was from The Binding Site (Saint Egrève, France). Peroxidase-conjugated anti-HK light-chain polyclonal antibody, nonconjugated anti-PK, and anti-FXII antibodies were from Enzyme Research. The secondary peroxidase-conjugated rabbit antigoat antibody was from Sigma (St Quentin-Fallavier, France). H-D-Pro-Phe-Arg-*p*NA substrate for PKa (L2120) was from Bachem (Weil-am-Rhein, Germany). Dextran sulfate (DS) and other chemicals were from Sigma.

### *In vitro* KKS reconstitution

Thawing and dilutions were done on ice in prechilled tubes with prechilled buffers. Excepting microBCA microplates, plasma and sodium dodecyl sulfate (SDS) containing tubes, all further dilutions or assays of proteins were done in polypropylene tubes or microplates coated 1–18 h at 0°C by 10 mg/ml bovine serum albumin in PBS (155.17 mM NaCl, 50 µM ZnCl_2_, 1.54 mM KH_2_PO_4_, 2.71 mM Na_2_HPO_4_, pH 7.2), rinsed once without albumin, then soaked but not dried and used within 1 h.

For gel analysis, FXIIa in one vial and PK and nHK in another one were diluted separately at 2× reaction concentration in PBS. Equal volumes of both solutions were mixed and samples were taken from 30 s to 30 min hereafter to be processed as below. Time-0 samples were taken before mixing. Assays were performed with concentrations within one order of magnitude around conditions originally chosen to set up other tests [16,17]: 2.5, 15, and 20.6 ng/µl (3.18 × 10^−8^ M, 1.70 × 10^−7^ M, 1.75 × 10^−7^ M) for FXIIa, PK, and nHK, respectively.

### Gel electrophoresis and western blot quantification

Samples were mixed with loading buffer with 20 mg/ml SDS and 25 mg/ml β-mercaptoethanol final concentrations then incubated at 80°C for 5 min and stored frozen. Polyacrylamide gel electrophoresis (SDS-PAGE) was performed using 120 mg/ml polyacrylamide gels prior to transfer onto nitrocellulose membrane (Hybond, GE HealthCare, Chalfont St. Giles, U.K.), followed by incubation with appropriate antibodies, then by enhanced chemiluminescence (ECL; Amersham, Arlington Heights, IL, U.S.A.). Densitometry quantification was conducted by means of a ChemiDoc™ XRS+ System camera and Image Lab™ software (Biorad, Hercules, U.S.A.) and expressed as % of cleavage.

### PKa reaction kinetics

For each of three independent experiments, the nHK substrate was diluted in 90% of the reaction volume of PBS, to a final concentration of 1.12 × 10^−7^ M, and two-, three-, four-, or five-times multiples and preincubated at 0°C or 37°C. PKa was added as a 10× concentration (1.6 × or 3.1 × 10^−8^ M final), mixed, incubated at 0°C or 37°C. One sample was taken immediately (T0 at ∼0 min), 1- and 2 min hereafter (T1, T2), and mixed with the same volume of 2× loading buffer, heated (80°C, 5 min) and frozen. Standard samples were taken prior adding the protease, their volume corrected by adding PBS (10% of final volume), and similarly processed. Gels were loaded with the same amount of nHK of each sample (S, around 135 ng per well) and five wells were loaded with twofold cascade dilutions of the nHK standard, (2S-S/8 range). Velocity, expressed in nM of nHK/s/nM of PKa, was calculated by dividing HK-cleavage amount during the first or second minute, by PKa concentration; nHK initial concentration was the theoretical one and not that obtained by the T0 deposit, substrate concentration at 1 min was the mean of both T1 deposits on the gel. For substrate consumption in the second minute of reaction, we used T1 concentration as the ‘initial concentration.’ Gels or data were discarded in conditions where the standard was not linear after removing up to one point, where both T1 quantifications differed by more than 20%, or where T0, T1, and T2 displayed abnormal or no differences. For each three independent experiment, at least five data pairs (substrate concentration, velocity) were selected to calculate *k*_cat_ and *K*_M_.

### FXIIa reaction kinetics

For each of four independent experiments, a precoated 96-well plate was prechilled, then wells were filled with 0 or 12.5–800 ng of PK diluted in 5 µl PBS (final concentration in 10 µl: 1.42 × 10^−8^ to 9.09 × 10^−7^ M, four wells each). At three different times, FXIIa (1 ng/5 µl, final concentration: 3.18 × 10^−8^ M) was sequentially added, in one well for each PK concentration, mixed by three up and down. The reaction was stopped with the same time sequence by adding 200 µl of 30°C prewarmed buffer (150 mM NaCl, 50 mM Tris, pH 7.8) with 10 ng/µl CTI, a specific FXIIa inhibitor, and 1 mM L2120 in all four wells of each PK concentration, using a four-tip comb. 1- to 10 s after adding substrate, FXIIa was added in the fourth well of each concentration as time zero control. PKa standard solutions were supplemented with the same substrate buffer. The plate was immediately introduced into a 30°C prewarmed spectrophotometer (Varioscan, ThermoFisher, Waltham MA, U.S.A.) and PKa activity monitored by absorbance at 405 nm (A_405_) for 30 min. Considering an A_405_ of 0.6 correspond to 0.1 mM of hydrolyzed L2120, only A_405_ values below 0.6 were considered not to exceed 10% of initial substrate consumption. A_405_ of PKa standard wells were corrected by the A_405_ of buffer without proteins. A_405_ of FXIIa containing wells were corrected for residual activity in presence of CTI by the A_405_ of their control well. PKa quantity was estimated using the standard and plotted against the time of incubation. FXIIa activity was calculated using the slope of the linear regression curve, and converted to initial velocity (in nM of PKa/s/nM of FXIIa).

### *K*_M_ and *k*_cat_ nonlinear determination

*K*_M_ and *k*_cat_ were first estimated using Hanes–Woolf linear regression, and then adjusted using nonlinear regression to minimize deviation between the data and the curve of velocity as a function of [S] using a calculation sheet (Excel software). Experiments were done three times for PKa, four times for FXIIa, and final *K*_M_ and *k*_cat_ were expressed as mean ± one standard deviation.

### Plasma samples

Plasma samples from healthy donors were processed according to French ethical policies governing the use of biological sample collection (Ministry of Health authorization DC-2008-634). Grenoble university hospital’s institutional review board (IRB South-East committee V) specifically approved the present study. Kinin-forming assay was performed as described [16].

### Mathematical models

We hypothesized the minimal KKS *in vitro* reconstitution as a two-step cascade of MM proteolytic enzymes, where ‘::’ indicates the enzyme–substrate complexes,

(E1) $$\{\begin{array}{cc} F12a\;+\;PK\;⇄\;F12a::PK\to \;F12a+PKa & \left(\text{Reaction}\;\;1\right)\\ PKa\;+\;nHK\;⇄\;PKa::nHK\to \;PKa+cHK & \left(\text{Reaction}\;\;2\right) \end{array}$$

In the MM model, the two steps in (eqn E1) were iteratively simulated by the following respective equations:

(E2) $$\{\begin{array}{cc} \frac{d[PK]}{dt}=-\frac{d[PKa]}{dt}=-k_{cat1}\frac{\left[F12a][PK\right]}{[PK]+K_{M1}}\;\; & \left(R1a\right)\\ \frac{d[nHK]}{dt}=-\frac{d[cHK]}{dt}=-k_{cat2}\frac{\left[PKa][nHK\right]}{[nHK]+K_{M2}} & \left(R2a\right) \end{array}$$

with initial conditions, [*PK*](*t* = 0) = [*PK*]_0_, [*nHK*](t = 0) = [*nHK*]_0_, [*cHK*](*t* = 0) = [*cHK*]_0_, [*PKa*](*t* = 0) = 0, and [*F*12*a*](*t* = 0) = [*F*12*a*]_0_, where [*X*] denotes the concentration of ‘X.’ The studied reaction displayed a faster start and a slower end rate than the MM model. The end of reaction rate was addressed by considering a competitive (Reaction 1) and an uncompetitive (Reaction 2) inhibition by cHK replacing (R1a) and (R2a) by:

(E3) $$\left\{ \begin{array}{cc} \frac{d[PK]}{dt}=-\frac{d[PKa]}{dt}=-k_{cat1}\times \frac{\left[F12a][PK\right]}{\left[PK]+K_{M1}/(1+[cHK]/KI_{1}\right)}\;\; & \left(R1b\right)\\ \frac{d[nHK]}{dt}=-\frac{d[cHK]}{dt}=-\frac{k_{cat2}}{1+[cHK]/KI_{2}}\times \frac{\left[PKa][nHK\right]}{\left[nHK]+K_{M2}/(1+[cHK]/KI_{2}\right)} & \left(R2a\right) \end{array}\right.$$

The reaction start rate was addressed by supplementing the initial conditions above with a presteady-state initial burst,

(E4) $$\{\begin{array}{c} \left[PKa](t=0)=inib\times [F12a\right]\\ \left[cHK](t=0)={\left[cHK\right]}_{0}+inib\times [F12a\right] \end{array}$$

where *inib*, *KI*_1_, and *KI*_2_ are unknown parameters.

Other models are described in Supplementary materials (SuppMat1). Equations were programmed using R software, ‘simecol’ and ‘ggplot2’ packages [18,19]: a 30-min reaction was simulated by 36000 step calculation using ‘lsoda’ solver. Results were expressed as the cleavage fraction of initial concentration. The goodness of the fit of the model was evaluated by the final sum of the squared distance (SSD) between theoretical curves and experimental data. An algorithm was devised to determine parameters (*inib*, *KI*_1_, *KI*_2_ …), which minimized SSD; iterations were stopped when the two first digits of each parameter did not change (deposited in Biomodels, MODEL2203210001 [20]). In general, there is often a time lag, Δt, between the real time of a reaction (theoretical time) and the observed experimental time, such as experimental time = theoretical time + Δt. This offset, Δt ≈ 44 s, obtained from another model (SuppMat1), was kept the same for all subsequent analyses. To evaluate the reliability of our model, Monte Carlo simulations were performed by randomly sampling 32 sets of data among our 32 KKS *in vitro* reconstitution assays.

## Results

### Human PKa and factor XII are active at 0°C

#### PKa reaction kinetics at 0°C

**Figure 1:**
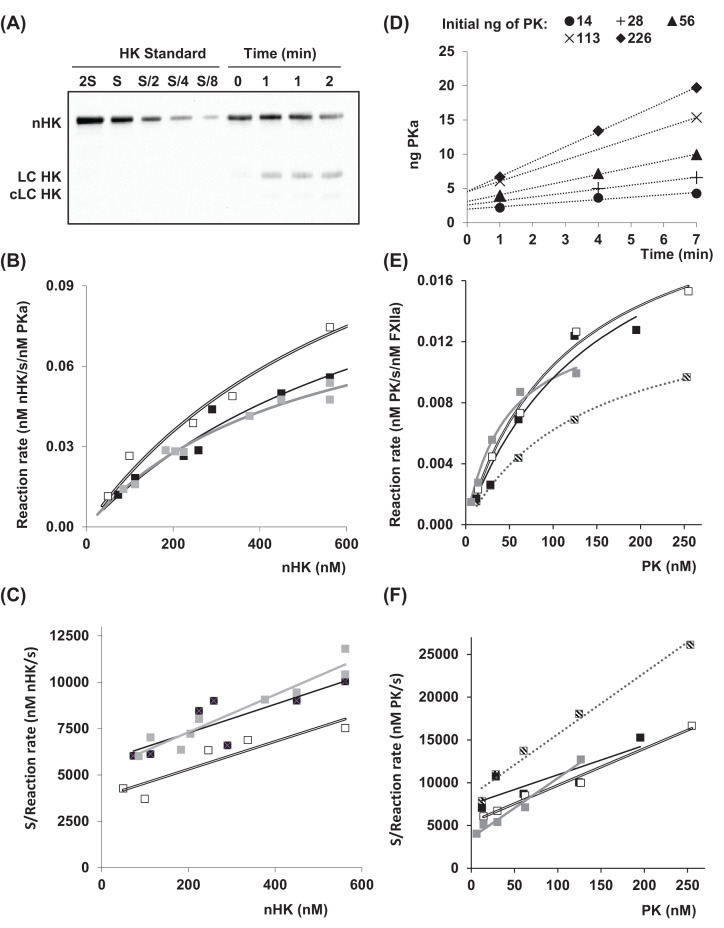
PKa and FXIIa kinetics determination (**A–C**) Kinetics of nHK cleavage by PKa at 0°C. (**A**) 5.6 × 10^−7^ M of nHK was incubated with 3.12 × 10^−8^ M of PKa at 0°C for 0–2 min. nHK standard (S = 135 ng) was used to calculate nHK cleavage for the reaction time. (**B**) Five western blots as displayed in (**A**) were performed with different nHK concentrations and velocity was plotted against nHK concentration for nonlinear regression. Representation of *n*=3 independent such experiments, displayed in white, gray, and black. (**C**) Same data as in (**B**), using Hanes–Woolf linearization. (**D–F**) Kinetics of PK activation by FXIIa at 0°C. (**D**) The indicated amount of PK was incubated with 1 ng FXIIa for 1–7 min at 0°C in 10 µL in a 96-well plate. Hydrolysis of L2120 was then monitored by A_405_ for 30 min at 30°C and compared with a PKa standard. (**E**) The slopes of the five linear regressions obtained in (**D**) were converted to initial velocity, and plotted against PK concentration for nonlinear regression. Representation of *n*=4 independent experiments, displayed in white, gray, hatched, black. (**F**) Same data as in (**E**), using Hanes–Woolf linearization.

We measured *k*_cat_ and *K*_M_ of PKa using nHK as substrate at 0°C, a temperature used in earlier KKS investigations [11,14,16] (Figure 1A–C) and at 37°C, as human blood temperature. Some methodology options were guided by initial settings of other tests of KKS exploration: an introduction of Zn-supplemented PBS, a high enzyme/substrate ratio, and an assessment of PKa activity by nHK cleavage (Figure 1A, SuppMat2), but not by BK release as in [12]. Table 1 shows kinetic parameters at 37°C, which have been found comparable to those observed at 25°C [12]. At 0°C, *k*_cat_/*K*_M_ showed values only five-times lower than at 37°C, indicating that PKa was active from 0°C to 37°C (Table 1).

**Table 1:** Kinetic parameters for HK cleavage by PKa, and PK activation by FXIIa

Enzyme/substrate	Temp. (°C)	*k*_cat_ (s^−1^)	*K*_M_ (nM)	*k*_cat_/*K*_M_ (s^−1^ × nM^−1^)
PKa/nHK	0	0.133 ± 0.034	672 ± 150	1.98 × 10^−4^
	*25 ^(a)^*	*0.63 ± 0.02*	*1380 ± 120*	*4.57* ×* 10^−4^*
	37	0.637 ± 0.241	1090 ± 200	5.87 × 10^−4^
FXIIa/PK	0	0.0119 ± 0.0027	115 ± 24	1.66 × 10^−4^
	*37 ^(b)^*	*1.03 (0.95–1.13)*	*1800 (1650–2000)*	*5.72* × *10^−4^*

Results are expressed as the mean ± SD of three independent assays. (a) From Tayeh et al. [12], (b) from Tankersley et al. [13] with confidence level in brackets instead of SD.

#### FXIIa reaction kinetics at 0°C

FXIIa kinetic parameters using PK as substrate have already been established at 37°C [13], but not at 0°C. Quantifying PK conversion into PKa by western blot provides outcome measurements of low accuracy. FXIIa kinetics was therefore followed by the amidase activity of the newly formed PKa using L2120 as substrate and an excess of PK (*cf*. 2.5). After limited contact between FXIIa and PK on ice, the reaction was slowed down by a 20-fold dilution and stopped by an addition of CTI as FXIIa inhibitor. We found that 5 mg/ml CTI nearly fully inhibited FXIIa, while PKa standard activity was decreased by less than 10% (not shown). Linearization of curves of PKa formation kinetics did not cross the origin of coordinates (Figure 1D). This can be explained by a presteady-state initial burst (discussed below) and/or by a nonimmediate stop of the FXIIa–PK reaction. The slopes were used to calculate the reaction velocity. Data of four independent experiments are shown in Figure 1E,F, with calculated parameters of FXIIa reaction kinetics within the same order as those of PKa (Table 1).

### *In vitro* reconstitution of KKS does not follow MM kinetics

**Figure 2:**
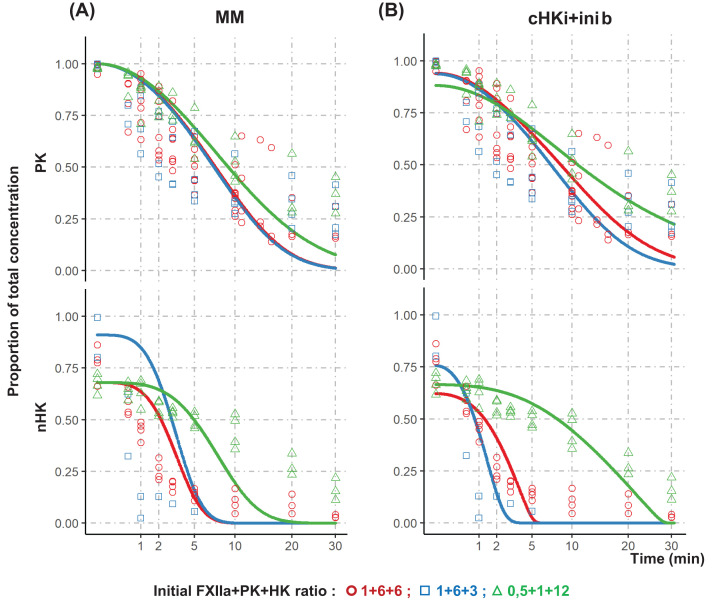
*In vitro* reconstitution of KKS and mathematical models Three different ratios (● 1/6/6, *n*=4 experiments; ■ 1/6/3, *n*=4; ▲ 0.5/1/12, *n*=3; with 1 corresponding to around 25 ng in 10 µl or 3 × 10^−8^ M) of FXIIa, PK, and HK were mixed on ice, and sampled from 0 to 30 min before SDS-PAGE and western blot analyses. The same data are displayed in both panels as the proportion of PK and native HK (nHK) initial concentration *versus* reaction time with a square-root scaling. (**A**) MM simulation of the reactions is displayed as plain lines of the corresponding color. (**B**) The cHKI+inib model includes an initial burst together with competitive and uncompetitive inhibitions of FXIIa and PKa, respectively, by cleaved HK (cHK).

A slow, but measurable, activity of both FXIIa and PKa enzymes on ice prompted us to follow the progression of the minimal proteolytic cascade made up of FXIIa, PK, and nHK and used in prior experiments [11]. The strategy was to build up a progress curve analysis of both reactions, using protein concentrations close to the plasmatic range (3–8 × 10^−7^ M each). The initial starting concentration set, called ‘1/6/6,’ was therefore ≈10^−7^ M PK and nHK, with a 1:1 ratio and a FXIIa concentration six-times lower, as assayed in prior experiments [11]. Progress curve analyses were performed to study variations within one order of magnitude of these initial concentrations, using 14 different sets of starting concentrations, three being displayed in Figure 2, the others in SuppMat3, for a total of 32 experiments. The reactions were stopped after different times by introducing aliquots into SDS-containing buffer, prior to western blot analysis and quantification.

#### KKS model only using MM simulation of both steps

Using the R software and ‘simecol’ library [18,19], a simulation of the two reactions was programmed using the MM equation, a simulation that assumes values of enzyme:substrate complex concentrations constant at each time lapse of the simulation, i.e. the ‘steady-state’ condition. This MM model (Figure 2A, SuppMat4A) differed from experimental results on two main points: a faster initial rate, both for PKa activation and nHK cleavage, and a slower rate at late times, for both reactions.

#### KKS initial burst model corrected half of the deviation found in MM model

To address the first issue (the faster initial rate), we considered the possibility of an initial burst: in this presteady-state condition, substrate consumption is faster than V_max_ of the Michaelian steady state, linked to a fast-initial turnover of the enzyme. Extension of the linear section of product formation *versus* time curve crossed the origin of *x*-axis at an ordinate that is proportional to enzyme concentration [21,22]. inib-1 and inib-2 identified the coefficients of proportionality for both FXIIa and PKa enzymes, respectively, while programming a double initial burst model (dIB) as described in the Materials and Methods and SuppMat1 sections.

We evaluated the quality of these models by SSD between experimental points and theoretical curves, and found that SSD values were nearly halved, between that observed from MM and dIB models, i.e. from 17.95 to 8.53 (SuppMat5, Model9V10). The initial burst coefficient of the first reaction was 0.39, as if 40% of FXIIa content rapidly activated one PK equivalent at the start of the reaction. The optimized value of this parameter was rather constant across all different models (SuppMat5).

In contrast, the second initial burst coefficient, calculated for nHK cleavage by PKa, was 1.64, considered as an implausible value, so we looked for more mechanistic models. First, since nHK and PK are complexed together in human plasma with a *K*_D_ ≈12 nM and in a ≈1:1 stoichiometry [23], we hypothesized a fast, non-Michaelian transition from PKa-nHK to PKa-cHK complex, i.e. *k*_Ct_ in M7 (SuppMat1). Second, we considered the three steps of the serine protease reaction, with both intermediate and acyl-enzyme-forming steps faster than the last one, a necessary condition for initial burst [5], i.e. *k*_e1_ and *k*_e2_ in M21, 24 (SuppMat1). These models gave unlikely values of the intermediate kinetic constants without improving the SSD-fitting criteria (SuppMat5).

The *in vitro* reaction on ice has been stopped by pipetting up and down sample in loading buffer at room temperature. A delay in stopping the reaction would account both for the initial faster rate in the progress curve analysis of both reactions and the intercept value in Figure 1C. If the stopping delay was the only modifying parameter of the MM model, it had to be long (i.e. 149 s) with a subsequent less fitted model than dIB (SSD = 9.49, M25V1; SuppMat5), excluding this issue as the only cause of the recorded fast initial rate of reactions. Combining initial burst as above and a stopping delay (SuppMat5, M27V2) led to a marginally improved fitting (SSD = 8.42) with three parameters (*inib1* = 0.30, *inib2* = 1.06, Δ*t* = 38 s). These values were realistic and could be, respectively, interpreted as (i) an initial burst in reaction 1; (ii) an immediate cleavage of one nHK molecule by one newly formed PKa equivalent, either by initial burst or by preferential cleavage within some pre-existing PKa-nHK association [23]; (iii) a delay between sampling the reaction and effective stop by the SDS buffer. We fixed *inib2* at its maximal value of 1, with a subsequent optimized time offset of 44 s (SuppMat5, M26V2). Thus, we suggest that a stopping delay and unidentified mechanisms of the initial burst accounted for the faster initial reaction velocity in the progress curve analysis.

#### Inhibition by the end product of cascade and slower end of reaction curves

We next considered the apparently slower reactions than predicted after the first minutes. Since there was no carrier protein in reaction buffer, a FXIIa protein amount could hypothetically bind onto plastic despite the coating procedure and thus became inactive. We did not detect diluted FXIIa inactivation on coated tubes, or after 30 min on ice. A 0-rate degradation reaction of FXIIa, combined with initial burst, would end with 25% of FXIIa activity only, after 30 min, to marginally improve the SSD (M29V1; SuppMat1,5). This ruled out a decay of FXIIa as the explanation of the slower end of reaction.

An inhibitory function of cHK, the end product of reaction, on both FXIIa and PKa enzymatic activities has been next hypothesized, which effectively fitted models to experimental data, lowering the SSD value from 8.43 (M26V2, SuppMat5) to 6.37 for competitive inhibition of FXIIa and uncompetitive inhibition of PKa (Figure 2C and Table 2). To rule out that these KI values are artefactually linked to the protein ratio and concentration range tested, 600 Monte Carlo simulations of Model26v2 were performed. Fitted values varied less than one order of magnitude, with unimodal distribution for SSD, *inib* and KI_1_ (Table 2, SuppMat6). While KI_2_ had a bimodal distribution, SSD for the second reaction was unimodal and all the three best fitted models involved uncompetitive inhibition of PKa by cHK (SuppMat5), so we excluded the performance of the inhibition model to be artefactually linked to the selections of tested initial concentrations.

**Table 2:** Performance of the mathematical models of KKS *in vitro* activation

Model	Parameter	Value	Dispersion median (2.5–97.5% range)	Expected value	Unit
MM	SSD	17.95	17.79 (11.48–25.56)		No unit
cHKI+inib	SSD	6.37	6.03 (4.63–7.53)	<8.42^(a)^	No unit
	inib	0.32	0.37 (0.21–0.53)	0–1	No unit
	KI1	250	219 (120–598)	1–1000	nM
	KI2	12.5	10.3 (7.2–15.9)	1–1000	nM

Models were first validated according to the lowering of the SSD between experimental data and the simulated curves. Parameter dispersions are from a Monte Carlo approach with *n*=600 simulations (SuppMat6). Abbreviations: MM, Michaelis-Menten simulation; cHKI+inib, simulation considering an initial burst (inib), a competitive (K_I1_), and uncompetitive (K_I2_) inhibition of FXIIa and PKa, respectively, by cHK.

(a) SSD value for a Model considering only the initial burst (SuppMat5).

It has not been possible to conclude on the inhibition mechanism (competitive, noncompetitive, or uncompetitive): the simulation gave similar SSD and KI-fitted values within the ranges of 11–200 and 175–400 nM against FXIIa and PKa (M26V1-9, SuppMat5). In order to decipher the interaction between cHK and both enzyme activities, we added cHK in the current experimental set up used for FXIIa reaction kinetics: cHK also alleviated CTI inhibition of FXIIa, preventing an effective end of PKa activation and data analysis. We also added four-times more cHK than nHK prior to measure PKa kinetics, but this blurred the cleavage evaluation of the initial nHK, beyond the low precision of a western blot quantification. We concluded that investigation of inhibition of KKS by cHK requires another experimental design.

### *Ex viv*o activation of KKS

**Figure 3:**
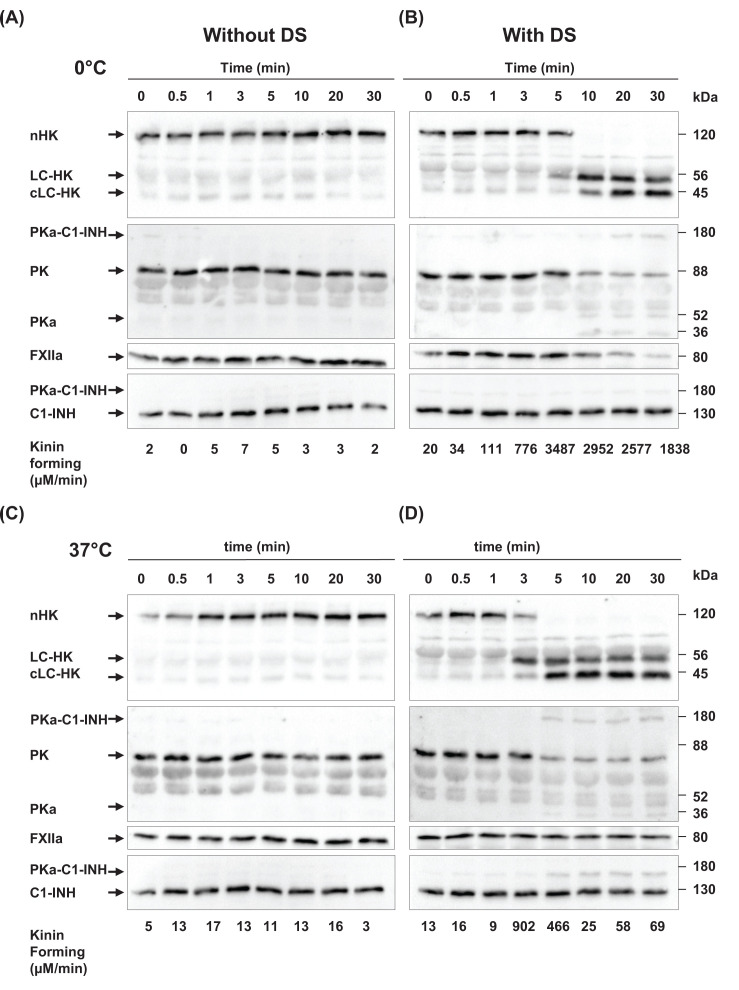
Activation of KKS in plasma Human plasma was thawed and activated (**B,D**) or not (**A,C**) with DS at 0°C (**A,B**) or 37°C (**C,D**). One aliquot was taken and frozen at indicated times in denaturing and reducing buffer, and analyzed by western blot for all the indicated proteins. Kinin-forming activities were measured from other aliquots according to [12]. Data of the figure are representative of three independent experiments. Abbreviations: C1-INH, C1 inhibitor, cLC-HK, LC-HK, and nHK, cleaved light chain, light chain, and native high-molecular-weight kininogen, respectively. FXIIa, activated FXII; PK, plasma PK; PKa, activated plasma kallikrein (52 and 36 kDa species); PKa-C1-INH, covalent kallikrein-C1-INH association.

We wanted to formally compare KKS activation between human plasma and the *in vitro* reconstitution. In condition of plasma activation by DS [14,16], FXIIa concentration is not fixed but resulted from a progressive transformation of FXII into FXIIa by DS and by PKa in a positive feedback loop. Furthermore, in plasma, both FXIIa and PKa are under the control of serpins, even if C1-INH is hypothetically considered as being inactive at 0°C [14]. Human plasma has been submitted to DS activation both at 0°C and 37°C and the molecular species and amidase activity of PKa using L2120 as substrate [16] have been followed. Figure 3 and SuppMat7 show the evolution of KKS molecular species and enzymatic activity.

As shown in Figure 3A,C, KKS proteins remained unmodified for 30 min at 0°C and at 37°C in the absence of DS. But in the presence of DS, a production of PKa and cHK is observed (Figure 3B,D), in a process that developed faster at 37°C than at 0°C, confirming data of Table 1. All proteins remained stable for 30 min without DS (Figure 3A,C). The higher kinin forming at 0°C than at 37°C may be due to a closer C1-INH control over the KKS proteases at 37°C [14]. The KKS control by serpin is shown by C1-INH-PKa complex formation at 37°C, as observed on Figure 3D. Unexpectedly traces were also shown at 0°C, suggesting that interaction between serine-protease and serpin slowly developed at 0°C and might explain a possible down-regulation of kinin forming after 5 min (Figure 3B).

Different modes of KKS activation mode have been investigated: DS *ex vivo* and functional FXIIa *in vitro*. A delay of *ex vivo* activation of PK in plasma (Figure 3B,D) was expected as compared with the *in vitro* experiment. Once started, a maximum PK activation (complete or not) occurred within a few minutes in Figure 3BD in line with data of Figure 2. Residual native PK was visible after 30 min, both at 0°C and at 37°C (Figure 3B,D). This might result *ex vivo* from FXIIa control by C1-INH, by other serpins or by α2-macroglobulin [4]. This feature is similar to the results observed *in vitro* at low FXIIa concentration (Figure 2A, green line) where PK was not fully activated after 30 min without any serpin control.

Whatever the mechanisms involved *in vitro* in experimental data illustrated by Figure 2A, the divergences between a minimal *in vitro* protease-zymogen cascade and its MM simulation (i.e. a preservation of zymogen PK and a fast complete nHK cleavage) were also found in *ex vivo* DS activation in human plasma samples (Figure 3B).

## Discussion

### Developing a progress curve analysis strategy with basic analysis tools

**Figure 4:**
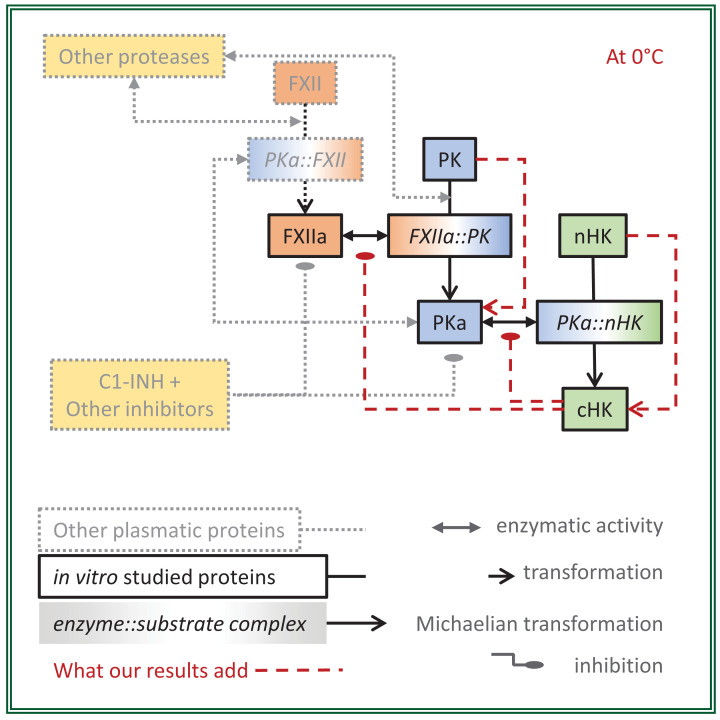
Schematic view of *ex vivo* activation of KKs at 0°C From minimal *in vitro* reconstitution (full color) of the human KKS (full and transparent color), FXIIa and KK display enzymatic activities five-times less on ice than at 37°C. The reaction kinetics of PKK and nHK cleavages differ from a MM simulation by a faster initial rate and a slower late rate, two features also found *ex vivo* in activated plasma (red dotted lines). Abbreviations: C1-INH, C1 inhibitor; cHK and nHK, cleaved and native forms of high-molecular-weight kininogen; FXII and FXIIa, factor XII and its activated form; PK and PKa, prekallikrein and plasma kallikrein.

With the present paper, we report *in vitro* and *ex vivo* data of the progression of the KKS proteolytic cascade by mixing FXIIa, PK, and nHK. Compared with *in vivo* situation, serpin control, and PKa-FXII-positive feedback loop were excluded, while maintaining a high enzyme/substrate ratio and using KKS natural substrates rather than peptide substrates. We developed a progress curve analysis, not an initial velocity study, and compared those results with two MM formatted differential equations. Methodology and findings are summarized in Figure 4.

Our experimental tools implied a low control of the stopping delay, variability in molecule quantification, and no measurement of kinetic constants such as *k*_on_ or *k*_off_ except global *K*_M_ and *k*_cat_. This lack of precision is congruent with the fact that any model should have far less optimized parameters than data, so we limit the models in order to introduce only one to three parameters to be optimized. A complete modeling of the interactions between one single molecule and a one-step enzyme requires a dozen kinetic constants [24]. A full description for the KKS would require even more, so we wonder if simple mechanisms could account for the early fast rate and slow end rate of the KKS cascade.

### About the initial burst in serine protease mechanism

Even if serine proteases are among the best known enzymes, the issue whether the acyl-enzyme formation or the final recovery of the functional enzyme is a rate-limiting step remained unsolved: earlier observations provided conflicting results, depending upon which small artificial peptide substrates were used [5]. Using natural substrates of FXIIa and PKa proteases, the strategy developed in the present study has been set up to give valuable argument pertaining with this issue. The observed initial burst cannot be formalized by an only faster acyl-enzyme formation. Likewise a nHK-PKa complex formation [23] followed by a fast transformation cannot by themselves explain the observed initial burst. Experimental errors in themselves cannot explain this too: even short sampling and mixing steps take time; no experimental setting could measure within the sampling tube the kinetic of SDS effect; mathematical simulations (SuppMat1) suggested an offset of nearly 44 s.

### About the mechanism of late reaction inhibition

Investigating with low protein concentration in polypropylene tube is a tricky condition, with subsequent protein adsorption and/or degradation. In place of carrier protein, coating of the reaction tubes is a good strategy to investigate KKS protein interaction prior to enzymatic reactions [25]. In these conditions, neither protease adsorption nor enzyme degradation could explain the slower rate of FXIIa and PKa kinetics in progress curve analyses. Inhibition by cHK was putatively a pertinent explanation, but may be not the only one. Inhibition by the end product of a metabolic pathway is not an uncommon phenomenon. In this respect, HK has been shown to be cleaved be platelet calpain [26], while inhibiting the same enzyme with a noncompetitive K_I_ of 5 mM [27]. This dual properties of nHK [28] and its long-lived complex interactions within KKS [29] require additional mechanistic explorations with more precise outcome than western blot.

Our model will thus be improved by implementing no optimized parameters for protease inhibition by cHK, but measured values of KI against FXIIa and PKa as well as their proven mechanism (competitive, uncompetitive, or noncompetitive). Likewise, with measured *k*_on_ and *k*_off_ parameters for the associations between these three KKS components, implication of their associations in the initial fast rate could be evaluated.

### Relevance of investigating KKS activity at 0°C

Data from human plasmatic enzyme activities at 0°C are scarce. Outside of KKS field, human glucose-6-phosphate dehydrogenase was found to display kinetics at 0°C [30] within one order of magnitude to that observed at 37°C [31]. Both records and the present data are in agreement with current cryobiology research [32].

Results of the present study demonstrate that PKa and FXIIa were active at 0°C with a *k*_cat_/*K*_M_ within the same magnitude order than at 37°C, while any remaining C1-INH are less functional. This could explain, why shipment of HAE-patient plasma samples at 0–4°C is definitely counterproductive for KKS analysis [33]. Our observations are congruent with a common recommendation for shipment of blood samples to laboratory for HAE diagnostic at +20°C within 48 h, and not 0°C [34].

### Relevance to plasmatic KKS activation

Even in the absence of explanation of the mechanism by which *in vitro* KKS reconstitution differed from MM kinetics, both initial burst and slower end reaction were also observed *ex vivo* in DS-activated plasma investigations, involving inhibitors and others proteases. *In vivo* KKS activation, in both physiological and pathological conditions (e.g. HAE) [7], is more complex, occurring in the continuous blood flow and in the presence of negatively charge activating surfaces. One can speculate that faster initial rate and slower late reaction are probably naturally selected features to develop a maximal local effect (BK release) and minimal global impact (PK preservation), a condition supposed to be a key characteristic of angioedema [7].

Developing assay able to distinguish between HAE with normal C1-INH and histamine-mediated angioedema is challenging. To this end, plasma activation by DS has been investigated using submaximal doses of the KKS activator in a 30-min incubation at 37°C [35]. Using a threshold cutoff based on the controls, HAE individuals presenting with normal C1-INH or idiopathic nonhistaminergic angioedema have been differentiated from histamine-mediated angioedema subjects. However, data from the present study suggest a consistent involvement of C1-INH in DS activation at 37°C; this condition is critical because of large differences of serpin function between samples, even from a single individual along time, could hamper a development of this assay.

Figure 3 underlines that kinin-forming test does not reflect full KKS activation [14,16]. C1-INH and maybe other serpins are less but still partially functional at 0°C. Furthermore, when testing a patient plasma sample, the full activation might not occur at the tested time. For blood protease cascades, such as thrombin formation, tests have been devised to follow the complete curve of activation and not only an end-point [36]. Analyzing the complete curve of cold KKS activation by DS requires a development of a low kinetic substrate, and these additions to the differential equations of our model (i) the hydrolysis of this new substrate and (ii) the residual inactivation at 0°C of both serpins by C1-INH. Such a model would provide valuable support for *ex vivo* investigation of KKS in samples from HAE patients and from other inflammatory conditions.

## Highlights

- Kinetics of nHK cleavage by PKa and of PK activation by factor XII display parameters of the same magnitude at 0°C and at 37°C.
- Kinetics of *in vitro* KKS activation and plasma activation by DS show a fast-initial rate and a slow late rate.
- MM simulation does not fit with *in vitro* KKS activation.

## Data Availability

R scripts used in the present paper are deposited in BioModels (19) and assigned the identifier MODEL2203210001, https://www.ebi.ac.uk/biomodels/MODEL2203210001. Intermediate scripts are freely available from the corresponding author upon request.

## Abbreviations

A_405_: absorbance at 405 nm
BK: bradykinin
C1-INH: C1 Inhibitor
cHK: cleaved species of high-molecular-weight kininogen
CTI: corn trypsin inhibitor
dIB: double initial burst
DS: dextran sulfate
FXIIa: activated factor XII
HAE: hereditary angioedema
HK: high-molecular-weight kininogen
KKS: kallikrein-kinin system
MM: Michaelis–Menten
nHK: native high-molecular-weight kininogen
PK: prekallikrein
PKa: (activated) plasma kallikrein
SDS: sodium dodecyl sulfate
SDS-PAGE: polyacrylamide gel electrophoresis under denaturing conditions
SSD: sum of the square distances between experimental points and theoretical curves
